# Advances and Innovations in Ablative Head and Neck Oncologic Surgery Using Mixed Reality Technologies in Personalized Medicine

**DOI:** 10.3390/jcm11164767

**Published:** 2022-08-16

**Authors:** Nadia Karnatz, Henriette L. Möllmann, Max Wilkat, Aida Parviz, Majeed Rana

**Affiliations:** Department of Oral and Maxillofacial Surgery, Heinrich Heine University Duesseldorf, 40225 Duesseldorf, Germany

**Keywords:** mixed reality, head neck tumor, surgical navigation, three-dimensional visual output

## Abstract

The benefit of computer-assisted planning in head and neck ablative and reconstructive surgery has been extensively documented over the last decade. This approach has been proven to offer a more secure surgical procedure. In the treatment of cancer of the head and neck, computer-assisted surgery can be used to visualize and estimate the location and extent of the tumor mass. Nowadays, some software tools even allow the visualization of the structures of interest in a mixed reality environment. However, the precise integration of mixed reality systems into a daily clinical routine is still a challenge. To date, this technology is not yet fully integrated into clinical settings such as the tumor board, surgical planning for head and neck tumors, or medical and surgical education. As a consequence, the handling of these systems is still of an experimental nature, and decision-making based on the presented data is not yet widely used. The aim of this paper is to present a novel, user-friendly 3D planning and mixed reality software and its potential application for ablative and reconstructive head and neck surgery.

## 1. Introduction

Optimal visualization of medical data in the treatment planning and decision-making of head and neck tumors, which provides targeted information, forms the basis for interdisciplinary communication. However, it is crucial to meet the different professional requirements of the various disciplines. The radiologist requires 2D imaging of the highest quality in order to detect or diagnose pathologies [1,2]. The surgeon bases his therapeutic decisions, in addition to his clinical experience, largely on radiological cross-sectional imaging. This provides him with pre- and intraoperative information, which, however, is not available to the pathologist. Specialties such as radiation therapy or oncology often still require their own imaging for planning or monitoring their therapy [3,4,5]. These monodisciplinary approaches significantly complicate communication. For example, traditional 2D cross-sectional imaging still serves as the basis for diagnostic and therapeutic decisions, although 3D models are already being calculated from 2D cross-sectional images using segmentation and rendering techniques to visualize regions of interest. However, conventionally, these 3D models are still static renderings projected onto a 2D plane to be visualized on a 2D screen [6]. A milestone in medicine has been the generation of a digital interface between 2D radiological slice imaging and the 3D surgical environment in computer-assisted surgery using a multiplanar view with colored 3D volume rendering of patients’ hard and soft tissue. By enabling the use of intraoperative computer-assisted navigation technology in the surgical site based on one or more imaging modalities. The optimized spatial orientation enables higher accuracy of surgical results as well as gentler surgical methods [7,8]. So far, this digital 2D/3D interface is still conditionally available to the surgeon. However, for adequate treatment of craniofacial tumors, computer-aided visualization and planning of 3D image data are necessary for all disciplines involved. For this purpose, an interdisciplinary image viewing interface would be required to visualize multimodal image data (computer tomography (CT), magnetic resonance imaging (MRI), cone beam-CT (CBCT), positron emission tomography (PET), PET/CT, and single photon emission computed tomography (SPECT)) for diagnostics, therapy planning, therapy sequence, treatment response, and search for distant metastasis or quality control [9,10]. Mixed reality (MR) technology, as a new tool in medicine and other disciplines, is a possible digital step in this direction. It is a new digital holographic visualization technology that allows virtual 3D objects to be created in space from radiological cross-sectional images, providing an immersive experience and possibilities for interactions with objects that would not be possible in a 2D environment. Therefore, MR merges the real world and virtual environments, creating a collective surrounding in which physical and digital objects are able to interact [11,12]. By combining the real world with the virtual world, medical data can be processed and visualized in real-time in a computer-generated environment [13].

Difficult-to-understand three-dimensional anatomy and geometry of the human skull require a high degree of spatial imagination, which is a difficult skill to acquire. With the help of MR technology, topographic considerations, complex structures, and pathological lesions can be visualized in a way that can be understood across disciplines by visualizing segmentation results with photo-realistic textures, trajectories, and annotations. In addition, the visualization of the 2D slice images in MR, which is still indispensable for the radiologist, is still possible and can be enriched with additional information such as tumor extension and safety distance for the surgeon, sampling locations for the pathologist, or infiltration of structures for the radiation therapist. Other computer-generated virtual viewing systems have been described in the literature, such as virtual reality (VR) and augmented reality (AR) [14,15]. In virtual reality, special software and hardware are used to simulate an artificial 3D environment that is completely detached from reality and creates an independent virtual environment. The disadvantage here is that the user cannot move freely in real space and is no longer aware of the real environment. AR extends a real environment (e.g., the surgical field) with computer-generated content. This provokes a 3D experience with a more integrated and sophisticated perspective of the patient’s condition. Multiple data from different categories (such as preoperative and/or intraoperative MRI, CT, etc.) can simultaneously be captured [16,17,18].

However, AR only adds virtual objects as additional information to the real environment, while MR technology overlays synthetic content in the real environment, making interactions with the virtual objects possible. Therefore, AR and VR should be considered only as partial solutions, such as their use in teaching [19,20]. 

MR technology provides a specific, language-independent, and multidisciplinary tool to enable radiologists, surgeons, oncologists, radiation therapists, and pathologists to visualize radiological imaging in a way that meets mono- and interdisciplinary requirements.

## 2. Materials and Methods

The aim of this work was to demonstrate and establish a novel, user-friendly, all-in-one 3D planning and reviewing mixed reality software (Brainlab AG, Munich, Germany) for ablative and reconstructive head and neck surgery and to clinically evaluate it.

Since 2021, the possibilities of using mixed reality technology for ablative/reconstruction surgery have been investigated at the University Hospital Düsseldorf, Clinic for Oral and Maxillofacial Surgery.

The use of this immersive technology in the scope of this work can be divided into the following four categories:

1. 3D visualization in preoperative imaging and planning;
2. tumor board—decision making and quality control platform;
3. patient-specific information;
4. education/surgical training.

### 2.1. Mixed Reality Technologies

#### 2.1.1. Hardware

One of the main challenges of mixed reality technology is to develop a user-friendly interface between the hardware and the user. In particular, the implementation of MR technology on a head-mounted display (HMD) allows the user to be mobile and independent of a workstation. Several hardware technologies are currently available for visualizing immersive mixed reality content. With handheld displays, the real environment can be captured through the use of cameras and linked to digital elements to add virtual content from any perspective. In the field of education and training, the use of handheld displays offers a promising addition to the possibilities used so far [21]. However, their use in surgery is not practical due to the manual positioning required still a lot is experimental nature. With HMDs capable of spatial computing and wireless transmission of the mixed reality content, surgeons can visualize the medical data and move freely in space. Sensors on the devices enable sensing of the physical environment for spatial computing used to automatically integrate holographic information into the real world [22,23]. Global Positioning System (GPS) for location, accelerometers, and ambient brightness meters can further support the mixed reality integration and can increase the immersiveness. Visual observations from cameras together with accelerometers and ambient brightness meters are used to track the head position and orientation in 6DoF. By continuous tracking of the environment and user position, mixed reality can create a more interactive and immersive experience. With special MR headsets already on the market, such as Google Glass, Microsoft HoloLens, or Magic Leap, digital content is projected in real-time on a small screen in front of the user’s eyes [24]. Real-time data transmission and handheld controllers facilitate user interaction. Moreover, worth mentioning are projector-based mixed reality systems that map digital content by projecting it onto organic shapes [13]. 

#### 2.1.2. Fundamentals of Visualization

For visualizing medical data in MR, DICOM (Digital Imaging and Communication in Medicine) datasets can be used, based upon which 3D models can be calculated automatically by algorithms and converted into polygons [25]. Rendering pipelines can then be employed to visualize the polygonal objects with appropriate textures on the HMDs. In order for the virtual objects to be congruent with the real environment in the user’s field of view, their position, viewing direction, and viewing angle must be detected for a correct overlay. This also requires very high accuracy in the surface mapping of the environment and the continuous monitoring of the user’s movement [26]. Thus, these registration steps are used to achieve alignment between physical and virtual information [27,28]. Various registration modalities such as cameras, inertial sensors, or mechanical systems are used in location technologies. Here, a trade-off between localization accuracy and complexity often has to be found. Mixed reality applications are mostly based on inside-out trackers attached to head-mounted displays. Since these trackers are based on visual features, degradation of image quality due to motion blur or illumination changes can lead to loss of location. Therefore, the combination of camera localization with other sensors such as inertial sensors can be performed [29,30]. Even the smallest deviations can lead to significant misregistration of virtual objects or so-called “jitter” effects [31,32]. Very high demands are placed on a medical system in terms of precision and reliability.

### 2.2. 3D Visualization in Preoperative Imaging and Planning

**Figure 1:**
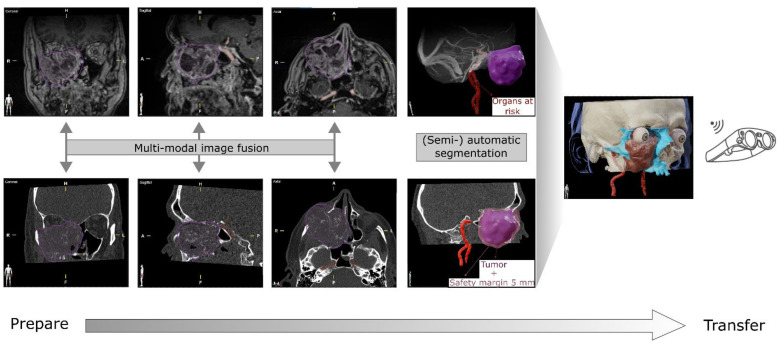
Planning steps for mixed reality visualizations. To fully leverage the potential of each imaging modality for surgical planning, the individual imaging series must be fused in a first step. Based on the resulting set of imaging data, the structures of interest can then be segmented automatically, or, in the case of pathological structures, semi-automatically and—if necessary—corrected manually. Due to the full integration of the mixed reality HMD (head-mounted-display) into the planning software, the segmented structures can then be transferred wirelessly to the devices and photo-realistically rendered.

The 3D visualization of head and neck tumors requires a high degree of representational accuracy of medical data [33]. The extent of a tumor as well as infiltration and destruction of structures influence treatment decisions. Mixed reality technology can be helpful as a visual interface between tomographic examination and spatial representation for surgical planning. It provides radiologists and surgeons with a multimodal interactive user interface for data processing and an efficient way to navigate through tomographic data, enabling surgical planning tailored to the patient [34,35,36]. Combined with mixed reality technology, digital patient-specific models can be created with high precision, enabling individualized treatment planning [37]. After the patient was scheduled for complex tumor resection, 3D digital reconstruction was performed using preoperative cross-sectional imaging. CT and MRI scans were used as the basis for 3D visualization of the tumor, with CT imaging performed in x-mm slices after peripheral injection of a contrast agent and modern low-dose (0.2–0.5 mSv) protocols. MRI datasets were acquired in 0.8-mm slices, using a 1.5 Tesla MRI scanner. To create the spatial representation, the datasets were imported into the Brainlab Elements™ planning application (Brainlab AG, Munich, Germany) in the standard Digital Imaging and Communications in Medicine (DICOM) format. For reproducible orientation of the anatomical structures, a symmetrical view of the data is required for the three-dimensional reconstruction; therefore, the CT slice is aligned in all dimensions (axial, coronal, sagittal multiplanar view) according to the horizontal Frankfurt midsagittal planes. Automatic image fusion of the CT and MRI datasets makes the entire multimodal information available to the practitioner. The superimposition of soft tissue and hard tissue imaging thus allows the extent of the tumor, as well as its soft tissue as well as bony infiltration of adjacent structures to be visualized in three dimensions. This technique of multimodal rigid image registration is based on mutual information and uses features such as multiple resolutions, intensity rebinning, and scaling in parameter space [8]. With the help of the planning software, automated segmentation of the anatomical structures and target tumor tissue from the image datasets was performed. This is based on an atlas-based algorithm that derives appropriate congruences between the patient and atlas datasets [38]. Furthermore, by adding voxels, semi-automatic segmentation is possible to improve the delineation of critical structures—this was performed using the radiological scans as a mapping aid. Selective segmentation also allows simulation of resection boundaries or reconstructions. The time required to create a 3D reconstruction depends on the complexity of the case. Cases with complex pathology and anatomy require extensive discussions led by the treating surgeons, the more complex the case the higher the manual workup. In particular, poor radiologic scans require manual reprocessing in contrast to cases with high-quality radiologic scans [39]. To improve visualization, voxel regions can be assigned specific color and opacity values, or supporting parameters such as textures or annotations can be used to mark or graphically represent structures [40,41]. By transmitting the processed treatment plans over a wireless network to a head-mounted MR device with the appropriate viewer software (Magic Leap 1, Plantation, Florida, USA; Viewer version 5.3, Brainlab AG, Munich, Germany), it is possible to view the radiological data as well as their reconstructions as 3D holograms (see Figure 1). 

The user is now able to manipulate the digital reconstructions to view patient anatomy and pathology from different perspectives. Through the additional planning of trajectories, the surgeon is now able, for example, to evaluate different possible accesses or biopsies preoperatively in 3D with high accuracy. For research purposes only, not released yet.

### 2.3. Tumor Board—Decision-Making and Quality Control Platform

Considering the complexity of oncological care in the head and neck region, a multidisciplinary team is essential for diagnosis and therapy decisions. This team is composed of oral and maxillofacial plastic surgeons, ear, nose, and throat physicians, radiation therapists, oncologists, pathologists, and radiologists. Here, the actual purpose of the multidisciplinary tumor board is to provide a joint decision-making sensibility but also a platform for quality control of treatment in terms of adherence to guidelines and evaluation of therapeutic outcomes. This includes ensuring a correct diagnosis, especially staging and treatment planning, but also coordination of care and management of complications [42,43,44]. It has been shown that the demands of the visualization of data and findings on the part of the specialist disciplines are often divergent and that joint communication is not always easy and can therefore take up a lot of time [45,46]. By implementing mixed reality software in the multidisciplinary tumor board (MDT), it is possible to streamline and centralize the oncological care of head and neck tumors, as it provides a language-independent, patient-centered, and flexible virtual platform for visualizing all information. In addition, available system resources can be superimposed in MR. 

Interaction with segmented objects, for example, repositioning of specific structures, can be an integral part of therapy planning and is already possible on desktop planning workstations. In order for a tumor board meeting to be held entirely in mixed reality, these functionalities must of course also be transferred to this format. The prototype software used in this work included this function and thus enabled the free-hand placement/movement of mixed reality objects such as anatomical structures or imported implants. In advance of the MR application during the tumor board, the segmentation of the tumor as well as the delineation of the safety distance should be performed by the radiologist and the surgeon, respectively. In particular, critical structures that make R0 resection difficult can be marked. With the help of 3D rendering and mixed reality, the information can now be shared with the individual participants of the multidisciplinary tumor board, thus providing a basis for discussion regarding treatment options. Especially in complex surgical cases, surgical interdisciplinary collaboration can be used to exchange information about the feasibility of the intervention or surgical approaches. The interaction with the hologram, however, also enables non-specialist disciplines or assistant physicians to gain a better understanding of the problem. Through virtual panels, the MDT radiologist is able to access individual 2D slice images without closing central information. This improves the user experience for all members of the MDT. 

Mixed reality is leading the way in terms of surgical and pathology coordination. By integrating computer-assisted surgery datasets into the mixed reality environment, a three-dimensional (voxel-based) dataset allows matching with macroscopic assessment of the extent of a head and neck tumor. A major problem in tumor resection (without navigation) is the naming and exact assignment of the anatomical three-dimensional position of the specimens. Intraoperative navigation enables reliable marking and assignment of histological specimens. This data can be stored and transmitted in DICOM format to provide patient-specific tumor information. This allows the clinical extension to be compared with the radiological extension. Tumor mapping can be presented to MDT participants in 3D rendered form, including all anatomical structures so that decisions about tumor respectability do not rely solely on radiological imaging [8,47]. Another advantage is that this allows information to be shared that is otherwise only available to the treating surgeon. Decisions, critical issues, etc. can be documented using annotations and can be made available for post-operative discussion using mixed reality. Similarly, it should be possible for the pathologist to share pathology reports, staining, or other relevant microscopy results, or for the practitioner to share photorealistic representation of the patient’s clinic in the MDT.

With the additional implementation of radiotherapy control, there is a decided basis for decision-making with regard to patient-specific therapy decisions in complex cases such as recurrences. Thus, using mixed reality technology, the room becomes a 3-dimensional shared computer screen with all members of the MDT, similar to an electronic medical record in real-time to share case information. 

A special aspect of this is the remote collaboration feature, which allows tumor board participants to be virtually co-present through real-time data exchange. This virtual co-presence allows MDT meetings to be more efficient and by means of modern network technology, such as the 5G standard, an immersive environment can still be created and real-time communication via audio chat features can be enabled.

Especially for patients who are not treated at large tumor centers, this enables individualized patient therapy, as physicians can exchange information with colleagues from tumor centers at any time via the remote collaboration function. With regard to digitalization in medicine, with the help of mixed reality technology and the accompanying information platform, collaboration in large academic medical networks in the care of oncological care of head and neck tumors is conceivable. With the help of intercom functions and avatars, resources can be centralized. MDTs with MR technologies improve accessibility, especially for clinicians who are not on-site. This promotes better patient-specific care as well as control of quality measures.

### 2.4. Patient-Specific Information 

**Figure 2:**
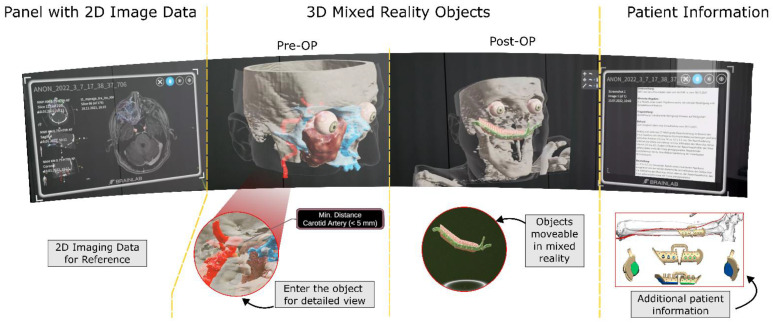
Capture of a mixed reality scene. Mixed reality has the ability to bundle all available information about the patient and make it accessible in a single, unified way. The conventional 2D images are still available and can also be examined in mixed reality. The 3D objects resulting from the 2D images can be interacted with, e.g., approaching the object can be used to understand anatomical positional relationships in detail. In addition, it is possible to integrate all other available patient information into the scene, for example radiological diagnoses, implant plans, or radiation therapy data. Kindly compiled by BrainLab.

Patient education not only serves to obtain informed consent from the patient prior to a surgical procedure but is also the basis for the exchange of information between physician and patient and should or can promote patient compliance during medical and rehabilitative treatment. However, informed consent can only be achieved if the patient is aware of the risks, the indication for the procedure, and the expected outcomes. Due to the complexity of surgical procedures in head and neck tumor surgery and increasingly shorter treatment times, information transfer is often difficult [48,49]. Nevertheless, ensuring adequate patient education regarding ethical and legal aspects is imperative. Thus, it has already been shown in the literature that increased patient satisfaction was achieved by adding additional information to the planned intervention in the form of paper-based or digital documents as well as audiovisual explanations [50]. However, an additional number of information sources also means a flood of information that may overwhelm the patient. Here, it is the physician’s task to place the multitude of information in an adequate medical context to avoid patient uncertainty. Personal doctor-patient contact is and remains essential. It is therefore imperative to convey targeted information to the patient in a compact and comprehensible manner in a conversation. Here, mixed reality technology as a digital medium can combine the human and communicative aspects of an informative patient conversation and patient-specific information on treatment. In the following, the possible use of mixed reality technology is described in more detail, which can be used for “diagnosis description” and therapy planning in the context of an adequate doctor-patient relationship in individual patient education [51]. The viewer of the head-mounted device serves as a platform for the display of digital information and objects. Here, the physician and patient see the same virtual objects. By visualizing the volumetric data from the patient’s own CT or MRI datasets in a 3D hologram, the patient is able to perceive his individual patient information and not just a description (see Figure 2).

The patient obtains the impression of looking into his own body, commonly referred to as his digital twin. Especially with regard to the complexity of head and neck tumors and the anatomy in this area, the patient is able to perceive his own disease for the first time, because he is not only shown a model of a head and neck tumor but his own tumor disease. By using annotations or segmented structures, the physician is able to explain patient-specific risks in addition to the general risks of a surgical procedure. This can significantly improve the understanding towards the extent of the surgery, possible postoperative complications, or functional limitations and help the patient accept them. Especially with regard to the importance of the safety distance of a head and neck tumor or the necessity of second or two-stage surgery, mixed reality technology is an adequate medium to explain these issues to the patient. The intuitive control of the system and the possibility of interaction with the virtual object allow doctors and patients to talk together about correlations or even alternatives. Thus, the patient is integrated into his patient education and not only “instructed”. This collaborative interaction between doctor and patient does not limit the “traditional” aspects of a patient education session such as interpersonal communication. By combining the real world with the aid of optical see-through glasses with virtual objects, medical data can be visualized and discussed with the patient. Nevertheless, the doctor is always available as a present and personal contact person. In particular, by integrating Standard Triangle Language (STL)—files of reconstructions in the context of rehabilitation of tissue defects, MR technology can clarify the complexity of the interventions [52]. Virtual annotations up to complex, photorealistic renderings can enhance the quality of targeted informative doctor-patient discussions. Thus, there is the possibility of common ground between doctor and patient.

### 2.5. Medical Education and Surgical Training

**Figure 3:**
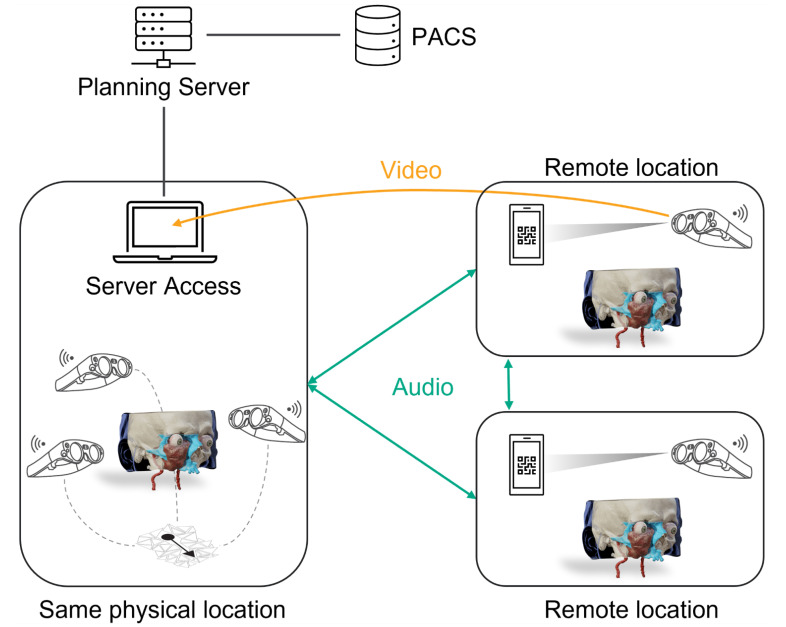
Schematic drawing of a (remote) mixed reality session. All required steps for the initial treatment planning are performed on a client PC, which accesses a server with the associated planning software installed. PACS integration facilitates access to the medical imaging data so that all information are directly available to the surgeon. Subsequently, multiple mixed reality HMDs at the same physical location as the client PC can connect to a joint session. Interaction with the virtual objects is synchronized in real-time, so that all participants see the same scenery from their viewpoints, thus enabling collaborative case discussions. Remote users can join the same mixed reality session by scanning a QR-code generated from and sent by the originator of the session to each remote participant. Bi-directional transmission of the audio signal enables communication across multiple locations via the HMD’s integrated microphone and speakers. User input synchronization and spatial audio enable immersive mixed reality experiences. In order to transmit the views from one session to other locations, the HMDs can send the video signal, which captures the real world enriched with the virtual objects, to the other participants via an integrated camera. In this way, also people who are not wearing an HMD can be involved in the session. Kindly compiled by BrainLab.

Mixed reality-based technology can open up new ways to teach medical content. The use of immersive experiences to facilitate the teaching and learning of complex subject matter enables resource-efficient teaching of theoretical and practical content [53]. The application of different extended reality technologies, including VR, AR, and MR is applicable to educating students as well as training physicians [54]. For example, extended reality technologies are currently being used in orthopedic computer-assisted surgery (CAS) systems and training simulators to increase surgical accuracy, improve outcomes, and reduce complications [55,56]. The use of MR technology to visualize a patient case was performed as part of a lecture for undergraduate students. Data from a head and neck tumor patient was used for this purpose. In addition to segmenting the tumor, anatomical structures such as eyes and skull bones were visualized to give students an impression of the location and extent of the mass. These can be shown or hidden separately or in various combinations. As part of the hands-on application, the CT scan was also shown in a separate panel and enabled a direct reference between 3D objects and their underlying 2D data in mixed reality. Communication between students and lecturers is also facilitated by annotation and structural textures. This enabled the visualization of patient-specific pathologies in addition to teaching macroscopic anatomy. With the help of the head-mounted device worn by the lecturer and students, the virtual model of the patient data scan could be discussed. In addition, by using a camera integrated into the head-mounted device, a surgeon is able to stream real-time videos of patients into the lecture hall and using the remote collaboration audio chat feature is able to communicate with the lecturer and the student to discuss the case. Thus, such a session can be projected onto a screen via live stream and display the patient and its associated 3D holograms (see Figure 3).

Depending on the question, it was thus possible to explain to the students the connection between theoretical knowledge and practical application. A recent study by Bork et al. 2018 investigated the application of AR technology in anatomy teaching. Participant feedback showed clear benefits in three-dimensional imagination compared to established teaching methods. Interactive applications and overall learning experience were also identified as clear benefits [57]. Another field of application for MR technology is its use in problem-based learning. Macroscopic anatomical knowledge forms the basis of all surgical training and continuing education. In preparation for planning a patient-specific implant after resection of a head and neck tumor, subjects should learn orbital reconstruction using a simulation tool to demonstrate the basis and intersections with computer-assisted surgery. Future studies to quantitatively evaluate the learning outcome are already being planned.

## 3. Results

Based on our previous experimental experience, it has been shown that MR technology is a suitable and accurate method for the visualization and treatment planning of head and neck tumors. Accurate radiological imaging of pathologies is an obligatory requirement for complex craniofacial interventions. In all presented areas, image data suitable for MR technologies was obtained and successfully processed. The time required for the processing of image data sets for the corresponding application fields, including data transfer, automatic and manual object segmentation, trajectories, and additional annotations, is highly variable and dependent on the complexity of the case. In particular, the processing of data in the preparation of an MR-based tumor board requires significantly more time. Before mixed reality technology can be used efficiently, exact requirements as well as the problem definition for the application area must be defined. This includes the requirements of the network environment to enable real-time data transmission as well as the specific content of the application areas as follows: When using MR technology in patient information, the automatic segmentation of CT data scans is sufficient since the aim here is merely to visualize pathologies in order to find a basis in the context of the patient conversation. Additional annotations can be set during the conversation, and prior manual processing of the data is only necessary in complex cases. The extent to which this new type of MR technology leads to increased understanding on the part of the patient or possibly to excessive demands, still needs to be clarified. Another limitation is that paper-based explanations remain obligatory due to the legal framework, so no added value can be expected in terms of time expenditure. In particular, the use of MR technology in the tumor board can be decisive, but the demands on technology and digitization are highest here. A time-consuming preparation of the data in line with the specialties is absolutely necessary in order to create a basis for decision-making. In particular, the implementation of the different protocols of the participating disciplines as well as their visualization requires more detailed investigations. Here, the segmentation and 3D rendering of the radiological CT scans for 3D visualization in preoperative imaging and planning is the most proven, as many of the workflow steps are already automated here with the help of the planning software. Investigating the transfer of teaching content through mixed reality in terms of efficiency and effectiveness must also be part of further investigations. Nevertheless, the literature has already identified an advantage to using VR and AR technologies. This allows the repetition of learning techniques while saving resources. Simulation is an important aspect of training, yet technology is not yet able to simulate haptic aspects of an examination or intervention [53,58,59]. Qualitatively, improvements in visualization and understanding can be expected in all application areas, as well as the facilitation of interdisciplinary communication in the future [60,61]. Aspects of quality control after processing of preoperative, intraoperative, and postoperative data are also conceivable sub-areas of mixed reality technology, as all data and results can be visualized for follow-up control. However, all application areas have in common that, so far, no defined workflow has been agreed upon to implement guidelines and legal requirements in a professional manner as well as to enable effective and efficient use of this technology.

## 4. Discussion

Various studies in oral and maxillofacial surgery have addressed potential applications of mixed reality technology in the visualization and treatment planning of head and neck tumors [62,63,64]. In this work, possible application scenarios of the new technology could be demonstrated. Modern imaging techniques such as CT and MRI are able to visualize parameters such as tumor volume and extent with high accuracy thanks to further developments in the last decades. This has led to an improvement in the staging of head and neck tumors [2,3]. However, there is still a need for a digital interface between tomographic examination and spatial imaging for surgical planning. MR technology, as a multimodal interactive image analysis platform, can create digital patient-specific 3D holographic models with high precision from multimodal image data [35,36]. Thus, by integrating segmented datasets, 3D visualization of tumor extent and clearance distance has the potential to provide better information on resectability or postoperative functional limitations. At the same time, 3D visualization from tumor mapping for comparison of radiological imaging with clinical parameters is possible [65]. Mixed reality technology in oncologic head and neck surgery increases reliability by visualizing safety distances and thus protecting vital structures. It can also serve as a planning aid in radiotherapy planning and assist in the planning process [62,66]. For postoperative follow-up, it is a useful tool to correlate outlined tumor margins and transfer them to different image datasets to detect tumor recurrence or the outcome of adjuvant chemotherapy and radiotherapy to evaluate treatment outcomes. Furthermore, the implementation of MR in a multidisciplinary tumor board allows the creation of a language-independent, patient-centered, and flexible virtual platform for visualization of all information. Important aspects of pre-, intra-, and postoperative treatment planning and quality control of the treatment strategy can be visualized and shared. In addition, available system resources can be coordinated [67,68]. Adequate patient education must consider anatomical and functional aspects. In this regard, MR technology is a viable tool for illustration and patient risk education. The planning software segments the anatomical structures with the automatic atlas-based algorithm and provides specific information about the patient’s clinical situation. The 3D visualization of the patient’s own disease and a patient-specific preoperative simulation can assist the patient in making treatment decisions. Studies showed that visual representation of information significantly improves the understanding of explanations [48,50]. Augmented reality, virtual reality (VR), and mixed reality (MR) can enable the delivery of medical content without negatively impacting patients in various medical disciplines. Yet, financial resources can be conserved, or ethical and regulatory constraints can be avoided [53,58]. 

## 5. Conclusions

The ideal application of MR technology would be a mobile or head-mounted display that allows the physician or operator to visualize patient data within the field of view rather than using one or more screens. Manipulation, simulation, and 3D holographic visualization of data can enable an increase in surgical accuracy and improve patient safety by reducing procedure-related complications. The broad range of applications also allows use of patient information, potentially resulting in increased patient compliance. In addition, technologies such as MR open the doors for the integration of novel learning methods into conventional medical teaching, thus initiating a paradigm shift towards active, student-centered learning with the help of multimodal, complementary learning methods. However, there have been few prospective, randomized studies comparing the benefits of using mixed reality technology in clinical practice with established methods in head and neck tumor surgery. Regarding the advantages of MR, this technology can play a major role in advanced head and neck cancer treatment.

## Data Availability

Not applicable.
